# Supplementary material for: The Evolution and Characterization of the RNA Interference Pathways in Lophotrochozoa
Source: Genome Biol Evol. 2024 May 7;16(5):evae098. doi: 10.1093/gbe/evae098 (PMC11114477; doi:10.1093/gbe/evae098)
Supplement: evae098_Supplementary_Data [file evae098_supplementary_data.zip › Supplementary_materials.zip!/selected_miRNAs/NoGe_selected_miRNAs/mirdeep_pdf_files/NMRB01000265.1_Notospermus_geniculatus_strain_Ushimado_scaffold265,_whole_genome_shotgun_sequence_8633.pdf]

Provisional ID : NMRB01000265.1_Notospermus_geniculatus_strain_Ushimado_scaffold265,_whole_genome_shotgun_sequence_8633
Score total : 29978.1
Score for star read(s) : 3.9
Score for read counts : 29969.7
Score for mfe : 1.5
Score for randfold : 0
Score for cons. seed : 3
Total read count : 58796
Mature read count : 57058
Loop read count : 0
Star read count : 1738

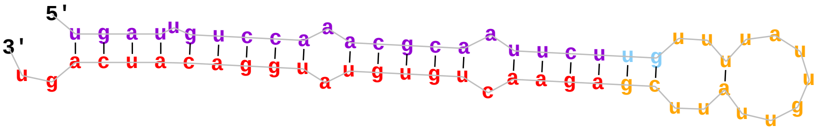

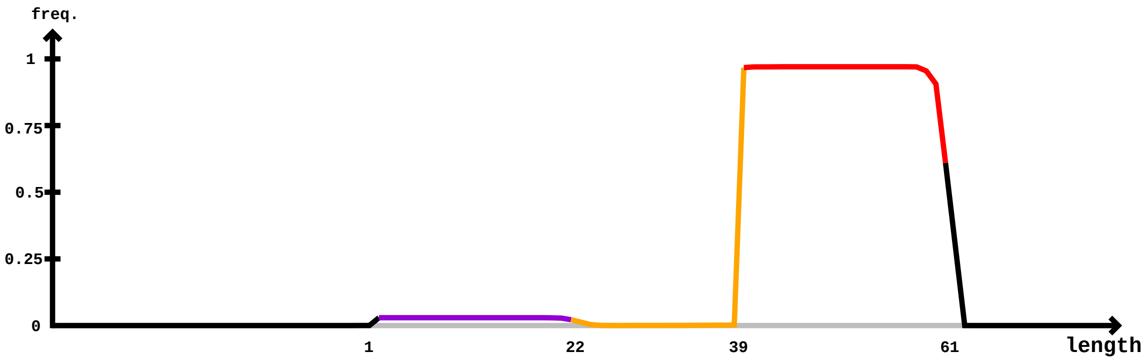


```
                                 Star                                  Mature
5'- gcaacuuccuucguaagaaagguuacaggcggugauuguccaaacgcaauucuuguuuuauuguuauucgagaacuguguauggacaucagugcuugccggccgguuuc   -3'   obs
    gcaacuuccuucguaagaaagguuacaggcggugauuguccaaacgcaauucuuguuuuauuguuauucgagaacuguguauggacaucagugcuugccggccgguuuc         exp
    .........(((....))).((((.((((((.((((.(((((.(((((.((((((..(.......)..)))))).))))).))))))))).))))))..)))).......   reads   mm   sample
    ...............................gugauuguccaaacgcaauucuu.......................................................   1   0   Fm1
    ...............................gugauuguccaaacgcaauucuuU......................................................   1   1   Fm1
    ................................ugauuguccaaacgcaUu...........................................................   1   1   Fm1
    ................................ugauuguccaaacgcaau...........................................................   3   0   Fm1
    ................................ugauuguccaaacgcaauu..........................................................   10   0   Fm1
    ................................ugauuguccaaacgcaauuc.........................................................   48   0   Fm1
    ................................ugauuguccaaacgcaaAuc.........................................................   1   1   Fm1
    ................................ugauuguccaaacgcaaCuc.........................................................   1   1   Fm1
    ................................ugauuguccaaacgUaauucu........................................................   1   1   Fm1
    ................................ugauuguccaaacgcaaAucu........................................................   4   1   Fm1
    ................................ugauuguccaUacgcaauucu........................................................   2   1   Fm1
    ................................ugauuguccaaacgcaaGucu........................................................   4   1   Fm1
    ................................ugauuguccaaacgcaauucu........................................................   402   0   Fm1
    ................................ugauuguccaaacgcaaCucu........................................................   2   1   Fm1
    ................................ugauuguccaaacgcaauucG........................................................   2   1   Fm1
    ................................Ngauuguccaaacgcaauucu........................................................   2   1   Fm1
    ................................uAauuguccaaacgcaauucu........................................................   1   1   Fm1
    ................................ugauuguccaaacgcaauucuu.......................................................   56   0   Fm1
    ................................ugauuguccaaacgcaCuucuu.......................................................   2   1   Fm1
    ................................ugauuguccaaacgcaauucuA.......................................................   3   1   Fm1
    ................................ugauuguccaaacgcaaCucuu.......................................................   1   1   Fm1
    ................................ugauuguccaaacgcaGuucuug......................................................   1   1   Fm1
    ................................ugauuguccaaacgcaaGucuug......................................................   1   1   Fm1
    ................................ugauuguccaaacgcaauucuug......................................................   82   0   Fm1
    ................................ugauuguccaaacgcaaAucuugu.....................................................   1   1   Fm1
    ................................ugauuguccaaacgcaauucuugu.....................................................   7   0   Fm1
    .................................gauuguccaaacgcaauucuug......................................................   2   0   Fm1
    .......................................................uuuuauuguuauucgagaacuguguauggacaucagu.................   1   0   Fm1
    ..........................................................uauuguuauucgagaacuguguauggacauca...................   1   0   Fm1
    ..............................................................guuauucgagaacuguguauggacaucag..................   1   0   Fm1
    .................................................................auucgagaacuguguauggaca......................   2   0   Fm1
    .................................................................auucgagaacuguguauggacauc....................   2   0   Fm1
    .................................................................auucgagaacuguguauggacaucag..................   1   0   Fm1
```

Star Mature

```
gcaacuuccuucguaagaaagguuacaggcggugauuguccaaacgcaauucuuguuuuauuguuauucgagaacuguguauggacaucagugcuugccggccgguuuc
.................................................................auucgaAaacuguguauggacaucag..................    1     1    Fm1
.................................................................auucgagaacuguguauggacaucagu.................    9     0    Fm1
.................................................................auucgagaacuguguauggacaucagugcuugccggccg.....    1     0    Fm1
.....................................................................Cagaacuguguauggacaucagu.................    1     1    Fm1
......................................................................agaacuguguauggacauc....................    86    0    Fm1
......................................................................agaacuguguauggacaCc....................    1     1    Fm1
......................................................................agaacuguguauggacaGc....................    1     1    Fm1
......................................................................agaacuguguauggacauU....................    3     1    Fm1
......................................................................agaacuguguauggacauca...................    88    0    Fm1
......................................................................agGacuguguauggacauca...................    1     1    Fm1
......................................................................Ggaacuguguauggacauca...................    1     1    Fm1
......................................................................agaacuguguauggacaucG...................    1     1    Fm1
......................................................................agaGcuguguauggacaucag..................    1     1    Fm1
......................................................................agaacuguguauggacGucag..................    1     1    Fm1
......................................................................Ugaacuguguauggacaucag..................    2     1    Fm1
......................................................................agaacuguguauggacaucaU..................    9     1    Fm1
......................................................................agaacuguguauggacaucag..................    507   0    Fm1
......................................................................agaacuguguauggacaAcag..................    2     1    Fm1
......................................................................agaacuguguauggacaGcag..................    3     1    Fm1
......................................................................agaacuguguCuggacaucag..................    1     1    Fm1
......................................................................agaacuguguGuggacaucag..................    1     1    Fm1
......................................................................agaacuguguauggacaCcag..................    2     1    Fm1
......................................................................agaacuguguaugAacaucag..................    1     1    Fm1
......................................................................agaacuguguauggacUucag..................    1     1    Fm1
......................................................................agaacuguguauggacCucag..................    2     1    Fm1
......................................................................Ugaacuguguauggacaucagu.................    1     1    Fm1
......................................................................Ngaacuguguauggacaucagu.................    1     1    Fm1
......................................................................agaacuguguauggacUucagu.................    5     1    Fm1
......................................................................agaacugugCauggacaucagu.................    1     1    Fm1
......................................................................agaacuguguGuggacaucagu.................    4     1    Fm1
......................................................................Ggaacuguguauggacaucagu.................    1     1    Fm1
......................................................................agaGcuguguauggacaucagu.................    1     1    Fm1
......................................................................agaacuAuguauggacaucagu.................    3     1    Fm1
......................................................................agaacuguguauggacaucagA.................    3     1    Fm1
......................................................................agaacuguguauggacCucagu.................    3     1    Fm1
......................................................................agaacuguguauggacaucGgu.................    1     1    Fm1
......................................................................agaacugCguauggacaucagu.................    1     1    Fm1
......................................................................agaacuguguauggacaucUgu.................    1     1    Fm1
......................................................................agGacuguguauggacaucagu.................    3     1    Fm1
......................................................................agaacuguguCuggacaucagu.................    5     1    Fm1
......................................................................agaacuguguauggacaucagG.................    21    1    Fm1
......................................................................agaacuguguauggacaGcagu.................    23    1    Fm1
......................................................................agaacuguguauggaUaucagu.................    1     1    Fm1
......................................................................agaacugGguauggacaucagu.................    1     1    Fm1
......................................................................agUacuguguauggacaucagu.................    1     1    Fm1
......................................................................aAaacuguguauggacaucagu.................    1     1    Fm1
......................................................................agaacuguguauggacauUagu.................    8     1    Fm1
......................................................................agaacuguguaugAacaucagu.................    1     1    Fm1
......................................................................agaacuguguauggacaCcagu.................    24    1    Fm1
......................................................................agaacuguguauggacaAcagu.................    15    1    Fm1
......................................................................agaacuguguUuggacaucagu.................    3     1    Fm1
......................................................................agaacuguguauggacaucagu.................    3568  0    Fm1
......................................................................agaacuguguauggCcaucagu.................    1     1    Fm1
......................................................................agaacuguguauggacGucagu.................    5     1    Fm1
......................................................................agaacuguguaCggacaucagu.................    1     1    Fm1
......................................................................agaacuguguauggacaucagC.................    1     1    Fm1
......................................................................agaacuguguauggacaucaguA................    4     1    Fm1
......................................................................agaacuguguauggacaucagAg................    5     1    Fm1
......................................................................agaacuguguauggacaucaguU................    18    1    Fm1
.......................................................................gaacuguguauggacaucagu.................    12    0    Fm1

..............................ggugauuguccaaacgcaauuc.........................................................    10    0    Fm2
................................ugauuguccaaacgcaCuuc.........................................................    1     1    Fm2
................................ugauuguccaaacgcaauuc.........................................................    15    0    Fm2
................................ugauuguccaaacgcaaCucu........................................................    2     1    Fm2
................................ugauuguccaaacgcaauucu........................................................    203   0    Fm2
................................ugauuguccaaacgcaauucG........................................................    2     1    Fm2
................................ugauuguccaUacgcaauucu........................................................    1     1    Fm2
................................ugauuguccaaacgcaaGucu........................................................    1     1    Fm2
................................ugauuguccaaacgcaauucuu.......................................................    11    0    Fm2
```

Star Mature

```
gcaacuuccuucguaagaaagguuacaggcggugauuguccaaacgcaauucuuguuuuauuguuauucgagaacuguguauggacaucagugcuugccggccgguuuc
................................ugauuguccaaacgcaaAucuu.......................................................   1     1    Fm2
................................ugauuguccaaacgcaauucuug......................................................   27    0    Fm2
................................ugauuguccaaacgcaauucuugu.....................................................   11    0    Fm2
.......................................................uuuuauuguuauucgagaacuguguauggacaucagu.................   8     0    Fm2
.......................................................uuuuauuguuauucgagaacuguguauggGcaucagu.................   1     1    Fm2
...........................................................auuguuauucgagaacGguguauggacaucagu.................   1     1    Fm2
...........................................................auuguuauucgagaacuguguauggacauGagu.................   1     1    Fm2
...........................................................auuguuauucgagaacuguguauggacaucagu.................   10    0    Fm2
......................................................................agaacuguguauggacauc....................   13    0    Fm2
......................................................................agaacuguguauggacauca...................   17    0    Fm2
......................................................................agaacuguguauggacCuca...................   1     1    Fm2
......................................................................agaacuguguauggacaAca...................   1     1    Fm2
......................................................................agaacuguguauggacaGca...................   1     1    Fm2
......................................................................agaacuguguauggacaGcag..................   2     1    Fm2
......................................................................agaacuguguauggacaucag..................   158   0    Fm2
......................................................................agaacuguguauggacaCcag..................   2     1    Fm2
......................................................................agaacuguguauggacaucagu.................   952   0    Fm2
......................................................................agaacuguguauggacaCcagu.................   5     1    Fm2
......................................................................agaacuguguauggacaAcagu.................   8     1    Fm2
......................................................................Ngaacuguguauggacaucagu.................   1     1    Fm2
......................................................................agaaUuguguauggacaucagu.................   1     1    Fm2
......................................................................agaacuguguauggaUaucagu.................   2     1    Fm2
......................................................................agaacuguguauggacCucagu.................   1     1    Fm2
......................................................................agaacuguguauggacaGcagu.................   9     1    Fm2
......................................................................agaacuguguauggacauUagu.................   1     1    Fm2
......................................................................agaacuguguauggacGucagu.................   4     1    Fm2
......................................................................agaacuguguCuggacaucagu.................   3     1    Fm2
......................................................................agaacuguguUuggacaucagu.................   1     1    Fm2
......................................................................agaacuguguauggacaucagG.................   5     1    Fm2
......................................................................Ugaacuguguauggacaucagu.................   2     1    Fm2
......................................................................agaacuguguauggacUucagu.................   2     1    Fm2
......................................................................agaacuguguauggacaucaguU................   32    1    Fm2
.......................................................................gaacuguguauggacaucagu.................   3     0    Fm2
.......................................................................gaacugugCauggacaucagu.................   1     1    Fm2

................................ugauuguccaaaUgcaauuc.........................................................   1     1    Mm1
................................ugauuguccaaacgcaauuc.........................................................   33    0    Mm1
................................ugauuguccaaacgcaauucu........................................................   61    0    Mm1
................................ugauuguccaaacgcaaCucu........................................................   1     1    Mm1
................................ugauuguccaaacgcaauucuu.......................................................   2     0    Mm1
................................ugauuguccaaacgcaauucuug......................................................   19    0    Mm1
................................ugauuguccaaacgcaaCucuug......................................................   1     1    Mm1
................................ugauuguccaaacgcaauucuuU......................................................   1     1    Mm1
................................ugauuguccaaacgcaauucuugu.....................................................   7     0    Mm1
......................................................................agaacuguguauggacau.....................   2     0    Mm1
......................................................................agaacuguguauggacauU....................   4     1    Mm1
......................................................................agaacuguguauggacauc....................   27    0    Mm1
......................................................................Ugaacuguguauggacauc....................   1     1    Mm1
......................................................................agaacuguguauggacauA....................   6     1    Mm1
......................................................................agaacuguguauggacCuca...................   1     1    Mm1
......................................................................agaacuguguGuggacauca...................   1     1    Mm1
......................................................................agaacuguguauggacauca...................   122   0    Mm1
......................................................................agaacuguguauggacaAcag..................   1     1    Mm1
......................................................................Ugaacuguguauggacaucag..................   1     1    Mm1
......................................................................agaacuguguauggacaCcag..................   2     1    Mm1
......................................................................agaacuguguauggacaucag..................   266   0    Mm1
......................................................................agaacuguguauggacaucaA..................   1     1    Mm1
......................................................................agaacuguguGuggacaucag..................   1     1    Mm1
......................................................................agaacuguguauggacaGcag..................   1     1    Mm1
......................................................................agaacuguguCuggacaucagu.................   3     1    Mm1
......................................................................agaacuAuguauggacaucagu.................   2     1    Mm1
......................................................................agaacuguguauggacauUagu.................   1     1    Mm1
......................................................................agGacuguguauggacaucagu.................   3     1    Mm1
......................................................................agaacuguguauggacaucaUu.................   9     1    Mm1
......................................................................agaacuguguauggacUucagu.................   7     1    Mm1
......................................................................agaacugugAauggacaucagu.................   2     1    Mm1
......................................................................agaacuguguauAgacaucagu.................   1     1    Mm1
......................................................................agUacuguguauggacaucagu.................   2     1    Mm1
......................................................................Ngaacuguguauggacaucagu.................   1     1    Mm1
......................................................................agaaGuguguauggacaucagu.................   1     1    Mm1
```

Star **Mature**

```
gcaacuuccuucguaagaaagguuacaggcggugauuguccaaacgcaauucuuguuuuauuguuauucgagaacuguguauggacaucagugcuugccggccgguuuc
......................................................................agaacuguguauggaUaucagu.................   1      1    Mm1
......................................................................agaacuguguauggacCucagu.................   6      1    Mm1
......................................................................agaacuguguauggacaAcagu.................   14     1    Mm1
......................................................................agaacCguguauggacaucagu.................   1      1    Mm1
......................................................................agaacuguguauggacGucagu.................   1      1    Mm1
......................................................................Ugaacuguguauggacaucagu.................   3      1    Mm1
......................................................................agaacuguguauggacaucagu.................   2970   0    Mm1
......................................................................agaacuguguauggacaGcagu.................   7      1    Mm1
......................................................................agaUcuguguauggacaucagu.................   1      1    Mm1
......................................................................agaacuguguauggaGaucagu.................   1      1    Mm1
......................................................................agaacuguguauggacaCcagu.................   16     1    Mm1
......................................................................agaacuguguUuggacaucagu.................   1      1    Mm1
......................................................................agaacugCguauggacaucagu.................   2      1    Mm1
......................................................................agaacuguguauUgacaucagu.................   2      1    Mm1
......................................................................agaacuguguauggacaucagA.................   12     1    Mm1
......................................................................agaacuguguauggacaucagG.................   2      1    Mm1
......................................................................agaCcuguguauggacaucagu.................   1      1    Mm1
......................................................................agaacuguguauggacaucaguU................   30     1    Mm1
......................................................................agaacuguguauggacaucaguA................   10     1    Mm1
.......................................................................gaacuguguauggacaucagu.................   5      0    Mm1

................................ugauuguccaaacgcaau...........................................................   1      0    Mm2
................................ugauuguccaaacgcaauu..........................................................   2      0    Mm2
................................ugauuguccaaacgcaauuc.........................................................   20     0    Mm2
................................ugauuguccaaacgcaauucu........................................................   81     0    Mm2
................................ugauuguccaaacgcaaCucu........................................................   2      1    Mm2
................................ugauuguccaaacgcaauucuu.......................................................   3      0    Mm2
................................ugauuguccaaacgcaauuGuug......................................................   1      1    Mm2
................................ugauuguccaaacgcaauucuug......................................................   11     0    Mm2
................................ugauuguccaGacgcaauucuug......................................................   1      1    Mm2
................................ugauuguccaaacgcaauucuugu.....................................................   2      0    Mm2
.......................................................uuuuauuguuauucgagaacuguguauggacauU....................   1      1    Mm2
...........................................................auuguuauucgagaacuguguauggacaucagu.................   1      0    Mm2
..............................................................guuauucgagaacuguguauggacaucag..................   1      0    Mm2
.................................................................auucgagaacuguguauggacauca...................   2      0    Mm2
.................................................................auucgagaacuguguauggacaucag..................   1      0    Mm2
.................................................................auucgagaacuguguauggacaucagu.................   1      0    Mm2
..................................................................uucgagaacuguguauggacaucag..................   2      0    Mm2
.....................................................................Cagaacuguguauggacauc....................   1      1    Mm2
.....................................................................Cagaacuguguauggacaucag..................   1      1    Mm2
......................................................................agaacuguguauggacau.....................   2      0    Mm2
......................................................................agaacuguguauggacGuc....................   1      1    Mm2
......................................................................agaacuguguauggacaAc....................   1      1    Mm2
......................................................................agaacuguguauggacauc....................   51     0    Mm2
......................................................................agaacuguguauggacauU....................   4      1    Mm2
......................................................................Cgaacuguguauggacauca...................   1      1    Mm2
......................................................................agaacuguguauggacauca...................   188    0    Mm2
......................................................................agaacuguguauggacaucU...................   3      1    Mm2
......................................................................agaacuguguauggacauUa...................   1      1    Mm2
......................................................................agaacuguguauggacaGca...................   1      1    Mm2
......................................................................agaacuguguauggacaCcag..................   5      1    Mm2
......................................................................agaacuguguauggacaAcag..................   2      1    Mm2
......................................................................agaacuguguauggacUucag..................   4      1    Mm2
......................................................................agaacuguguauggacGucag..................   1      1    Mm2
......................................................................agaacuguguauggacaGcag..................   4      1    Mm2
......................................................................agaacuguguUuggacaucag..................   2      1    Mm2
......................................................................agaacuguguauggacaucGg..................   2      1    Mm2
......................................................................agaacNguguauggacaucag..................   1      1    Mm2
......................................................................agaacuguguauggacaucag..................   896    0    Mm2
......................................................................agaacuguguauggacaucaU..................   3      1    Mm2
......................................................................agaacuguguauggacCucag..................   1      1    Mm2
......................................................................agaacuguguGuggacaucag..................   1      1    Mm2
......................................................................agaacuguguauUgacaucagu.................   1      1    Mm2
......................................................................agaacuguguCuggacaucagu.................   2      1    Mm2
......................................................................agaacugCguauggacaucagu.................   1      1    Mm2
......................................................................agaacuguguauggacaAcagu.................   12     1    Mm2
......................................................................agaacuguUuauggacaucagu.................   2      1    Mm2
......................................................................agGacuguguauggacaucagu.................   1      1    Mm2
......................................................................agaacuguguauggacaGcagu.................   6      1    Mm2
......................................................................Ugaacuguguauggacaucagu.................   1      1    Mm2
```

```
                                Star                             Mature
gcaacuuccuucguaagaaagguuacaggcggugauuguccaaacgcaauucuuguuuuauuguuauucgagaacuguguauggacaucagugcuugccggccgguuuc
......................................................................agaacuguguauggacauUagu.................   3      1    Mm2
......................................................................agaacuguguauggacaucagu.................   2175   0    Mm2
......................................................................agaacuguguauggacaCcagu.................   10     1    Mm2
......................................................................agaacuguAuauggacaucagu.................   2      1    Mm2
......................................................................agaacuguguauggacauAagu.................   1      1    Mm2
......................................................................agaacuguguauggacaucaUu.................   1      1    Mm2
......................................................................agaacuguguauggacGucagu.................   2      1    Mm2
......................................................................agaacuNuguauggacaucagu.................   1      1    Mm2
......................................................................agaacuguguauggacaucagC.................   2      1    Mm2
......................................................................agaacuguguauggacCucagu.................   3      1    Mm2
......................................................................agaacuguguUuggacaucagu.................   1      1    Mm2
......................................................................agaacuguguauggacaucagG.................   5      1    Mm2
......................................................................agaacuguguauggacUucagu.................   2      1    Mm2
......................................................................agaacuCuguauggacaucagu.................   1      1    Mm2
......................................................................agaUcuguguauggacaucagu.................   1      1    Mm2
......................................................................agaacuguguauggacaucagA.................   5      1    Mm2
......................................................................agaacuguguGuggacaucagu.................   4      1    Mm2
......................................................................agaacuguguaCggacaucagu.................   2      1    Mm2
......................................................................agaacuAuguauggacaucagu.................   1      1    Mm2
......................................................................agaacuguguauggacaucaguA................   4      1    Mm2
......................................................................agaacuguguauggacaucaguU................   13     1    Mm2
.......................................................................gaacuguguauggacaucagu.................   5      0    Mm2
.........................................................................acuguguauggacaucag..................   1      0    Mm2
.........................................................................acuguguauggacaucagu.................   3      0    Mm2
..........................................................................cuguguauggacaucagu.................   2      0    Mm2

..............................ggugauuguccaaacgcaauuc.........................................................   1      0    Fm3
................................ugauuguccUaacgcaau...........................................................   1      1    Fm3
................................ugauuguccaaacAcaau...........................................................   1      1    Fm3
................................ugauuguccaaacgcaau...........................................................   13     0    Fm3
................................ugauuguccaaacgcaaA...........................................................   1      1    Fm3
................................ugauuguccaCacgcaauu..........................................................   1      1    Fm3
................................ugauuguccaaacgcaauA..........................................................   10     1    Fm3
................................ugauuguccaaacgcaaCu..........................................................   1      1    Fm3
................................ugauuguccaaacgcaauu..........................................................   50     0    Fm3
................................ugauuguccaaacgcaaGuc.........................................................   1      1    Fm3
................................ugauuguccaaacgcaUuuc.........................................................   2      1    Fm3
................................ugauuguccaaacgcaaAuc.........................................................   4      1    Fm3
................................ugauuguccaaacgcaauuc.........................................................   181    0    Fm3
................................Agauuguccaaacgcaauuc.........................................................   1      1    Fm3
................................ugauuguccaaacgcaaCuc.........................................................   3      1    Fm3
................................ugaAuguccaaacgcaauuc.........................................................   1      1    Fm3
................................ugauuguccaaacgcaaGucu........................................................   3      1    Fm3
................................ugauuguccaaacgcaaCucu........................................................   1      1    Fm3
................................ugauuguccaUacgcaauucu........................................................   1      1    Fm3
................................ugauuguccGaacgcaauucu........................................................   1      1    Fm3
................................ugauuguccaaacgcaauucu........................................................   124    0    Fm3
................................ugauuguccaaacgcaauucuu.......................................................   6      0    Fm3
................................uNauuguccaaacgcaauucuug......................................................   1      1    Fm3
................................ugauuguccGaacgcaauucuug......................................................   1      1    Fm3
................................ugauuguccaaacgcaauucuug......................................................   43     0    Fm3
................................ugauuguccaaacgcaauucuugu.....................................................   2      0    Fm3
.................................gauuguccaaacgcaauucuug......................................................   1      0    Fm3
.............................................................uguuauucgagaacuguguauggacaucagu.................   1      0    Fm3
................................................................uauucgagaacuguguauggacaucagu.................   3      0    Fm3
.................................................................auucgagaacuguguauggacauca...................   2      0    Fm3
..................................................................uucgagaacuguguauggacauc....................   3      0    Fm3
...................................................................ucgagaacuguguauggacau.....................   8      0    Fm3
...................................................................ucgagaacuguguauggacauc....................   8      0    Fm3
...................................................................ucgagaacuguguauggacauU....................   7      1    Fm3
...................................................................ucgagaacuguguauggacauca...................   3      0    Fm3
.....................................................................Cagaacuguguauggacauc....................   2      1    Fm3
.....................................................................Cagaacuguguauggacaucag..................   3      1    Fm3
.....................................................................Cagaacuguguauggacaucagu.................   10     1    Fm3
......................................................................agaacuguguauggacau.....................   20     0    Fm3
......................................................................agaacuguguauggacCuc....................   1      1    Fm3
......................................................................agaacuguguauggacauc....................   494    0    Fm3
......................................................................agaacuguguauggacaCc....................   2      1    Fm3
......................................................................agaacuguguauggacUuc....................   1      1    Fm3
......................................................................agaacuguguauggacaAc....................   2      1    Fm3
```

Star **Mature**

gcaacuuccuucguaagaaagguuacaggcggugauuguccaaacgcaauucuuguuuuauuguuauucgagaacuguguauggacaucagugcuugccggccgguuuc			
....agaacuguguauggacauA....................	37	1	Fm3
....agaacuguguauggacauU....................	23	1	Fm3
....agaacuguUuauggacauc....................	1	1	Fm3
....agaacuguguCuggacauc....................	2	1	Fm3
....agaacuguguaGggacauca...................	1	1	Fm3
....agaacuguguauggacaGca...................	7	1	Fm3
....agaacuguguauggacGuca...................	5	1	Fm3
....agaacuguguauggacaCca...................	17	1	Fm3
....Ugaacuguguauggacauca...................	2	1	Fm3
....agaacuguguauggacaucU...................	21	1	Fm3
....agaacuguguauggacUuca...................	3	1	Fm3
....agaacuguguauggacauUa...................	1	1	Fm3
....agaacugAguauggacauca...................	1	1	Fm3
....agaacuAuguauggacauca...................	1	1	Fm3
....agaacuguguauUgacauca...................	2	1	Fm3
....agaacuguguUuggacauca...................	1	1	Fm3
....agaacuguguGuggacauca...................	2	1	Fm3
....Ngaacuguguauggacauca...................	1	1	Fm3
....agaacuguguauggacauca...................	2144	0	Fm3
....agaacuguguauggacaAca...................	5	1	Fm3
....agaacNguguauggacauca...................	1	1	Fm3
....agaacuguguaCggacauca...................	1	1	Fm3
....agaacugCguauggacauca...................	1	1	Fm3
....agaacuguguauggacCuca...................	5	1	Fm3
....agaacuguguauggacaucC...................	1	1	Fm3
....agaacuguguauggaUaucag..................	5	1	Fm3
....agaacuguUuauggacaucag..................	2	1	Fm3
....agaacuguguaugUacaucag..................	3	1	Fm3
....agaacuguguauggacauGag..................	2	1	Fm3
....Cgaacuguguauggacaucag..................	2	1	Fm3
....agaacuguguauggacauUag..................	6	1	Fm3
....agaacuguguauggacaucaU..................	38	1	Fm3
....agaacuguguauggacaucUg..................	2	1	Fm3
....agGacuguguauggacaucag..................	4	1	Fm3
....agaacuguguGuggacaucag..................	26	1	Fm3
....agaacuguguauggaGaucag..................	3	1	Fm3
....agaacuguguCuggacaucag..................	20	1	Fm3
....Ngaacuguguauggacaucag..................	4	1	Fm3
....aAaacuguguauggacaucag..................	6	1	Fm3
....agaUcuguguauggacaucag..................	1	1	Fm3
....agaacuNuguauggacaucag..................	1	1	Fm3
....agaacugGguauggacaucag..................	1	1	Fm3
....agaacuguguauUgacaucag..................	3	1	Fm3
....agaacuUuguauggacaucag..................	2	1	Fm3
....agaacuguguauggacaGcag..................	75	1	Fm3
....aNaacuguguauggacaucag..................	1	1	Fm3
....agaacuguCuauggacaucag..................	1	1	Fm3
....agaCcuguguauggacaucag..................	1	1	Fm3
....agaacuguguauggacUucag..................	24	1	Fm3
....agaacNguguauggacaucag..................	2	1	Fm3
....agaaAuguguauggacaucag..................	2	1	Fm3
....agaacuguguaugAacaucag..................	5	1	Fm3
....agaacCguguauggacaucag..................	1	1	Fm3
....agaacuguguauggacGucag..................	12	1	Fm3
....agaaUuguguauggacaucag..................	1	1	Fm3
....agaacugugAauggacaucag..................	1	1	Fm3
....agaacuguguauggaAaucag..................	2	1	Fm3
....agaaGuguguauggacaucag..................	2	1	Fm3
....agaacugugGauggacaucag..................	1	1	Fm3
....aUaacuguguauggacaucag..................	1	1	Fm3
....agaacuguguaAggacaucag..................	1	1	Fm3
....agaacuguguauggacaucag..................	13868	0	Fm3
....agaacuguguauggacaCcag..................	84	1	Fm3
....agaacuguguaugCacaucag..................	1	1	Fm3
....agaacuguguauAgacaucag..................	5	1	Fm3
....Ggaacuguguauggacaucag..................	2	1	Fm3
....agaacugugCauggacaucag..................	10	1	Fm3
....agUacuguguauggacaucag..................	2	1	Fm3
....agaacuguAuauggacaucag..................	1	1	Fm3
....Ugaacuguguauggacaucag..................	13	1	Fm3

Star **Mature**

gcaacuuccuucguaagaaagguuacaggcggugauuguccaaacgcaauucuuguuuuauuguuauucgagaacuguguauggacaucagugcuugccggccgguuuc			
....agaacuguguauggacaucGg..................	3	1	Fm3
....agaacuguguUuggacaucag..................	12	1	Fm3
....agaacuguguauggacCucag..................	22	1	Fm3
....agaacuguguauggacaucCg..................	2	1	Fm3
....agaacugCguauggacaucag..................	2	1	Fm3
....agaacuguguauggGcaucag..................	5	1	Fm3
....agaacuguguauggUcaucag..................	1	1	Fm3
....agaacuguguauggCcaucag..................	2	1	Fm3
....agaacuguguauggacaAcag..................	58	1	Fm3
....agaGcuguguauggacaucag..................	2	1	Fm3
....agaacuguguaugAacaucagu.................	3	1	Fm3
....agaacuguguauggacCucagu.................	32	1	Fm3
....agaacuguguauggacauAagu.................	5	1	Fm3
....Ggaacuguguauggacaucagu.................	3	1	Fm3
....agaacuguguauggacUucagu.................	26	1	Fm3
....agaGcuguguauggacaucagu.................	2	1	Fm3
....agaacuguguauCgacaucagu.................	5	1	Fm3
....agaacNguguauggacaucagu.................	4	1	Fm3
....agaacuguguUuggacaucagu.................	20	1	Fm3
....agaacuguguauAgacaucagu.................	2	1	Fm3
....agaacuNguguauggacaucagu.................	2	1	Fm3
....Cgaacuguguauggacaucagu.................	1	1	Fm3
....agaacuguguGuggacaucagu.................	22	1	Fm3
....agaaAuguguauggacaucagu.................	1	1	Fm3
....agaaUuguguauggacaucagu.................	5	1	Fm3
....agaacuUuguauggacaucagu.................	1	1	Fm3
....agaacuguguauggaUaucagu.................	2	1	Fm3
....Ugaacuguguauggacaucagu.................	17	1	Fm3
....agaacuguAuauggacaucagu.................	4	1	Fm3
....agaacuguguauggacaucagC.................	1	1	Fm3
....agaacuguguauggacaucGgu.................	2	1	Fm3
....agaacuguUuauggacaucagu.................	1	1	Fm3
....agaacuguguauggacauGagu.................	1	1	Fm3
....aAaacuguguauggacaucagu.................	8	1	Fm3
....agaacAguguauggacaucagu.................	3	1	Fm3
....agaacuguguauggacGucagu.................	23	1	Fm3
....agaacuguguauggacaucaUu.................	13	1	Fm3
....agaacuAuguauggacaucagu.................	3	1	Fm3
....agaacuguguauggacauUagu.................	11	1	Fm3
....agaacuguguaugCacaucagu.................	5	1	Fm3
....agaacuguguauggacaucaAu.................	4	1	Fm3
....agaacuguguauggGcaucagu.................	5	1	Fm3
....Ngaacuguguauggacaucagu.................	6	1	Fm3
....agaacuguguaugUacaucagu.................	9	1	Fm3
....agaacuguguaCggacaucagu.................	4	1	Fm3
....agaCcuguguauggacaucagu.................	2	1	Fm3
....agaacuguCuauggacaucagu.................	3	1	Fm3
....agaacuguguauggaAaucagu.................	1	1	Fm3
....agaacugugGauggacaucagu.................	1	1	Fm3
....agaacuguguauggUcaucagu.................	4	1	Fm3
....agUacuguguauggacaucagu.................	1	1	Fm3
....agaacugAguauggacaucagu.................	2	1	Fm3
....agGacuguguauggacaucagu.................	8	1	Fm3
....agaacuguguaAggacaucagu.................	2	1	Fm3
....agaacuguguCuggacaucagu.................	19	1	Fm3
....agaacuguguauggacaucagu.................	18758	0	Fm3
....agaacuguguauggacaucaCu.................	1	1	Fm3
....agaUcuguguauggacaucagu.................	1	1	Fm3
....agaacuguguauggacaucagA.................	34	1	Fm3
....agaacugugAauggacaucagu.................	1	1	Fm3
....agaacuguguauggacaucCgu.................	2	1	Fm3
....agaacuguguauggaGaucagu.................	1	1	Fm3
....agaacuguguauggCcaucagu.................	2	1	Fm3
....agaacugCguauggacaucagu.................	4	1	Fm3
....agaacGguguauggacaucagu.................	1	1	Fm3
....agaacuguguauggacaGcagu.................	86	1	Fm3
....agaacuguguauUgacaucagu.................	3	1	Fm3
....agaacuguguauggacaAcagu.................	81	1	Fm3
....agaacuguguauggacaCcagu.................	95	1	Fm3
....aUaacuguguauggacaucagu.................	4	1	Fm3

Star Mature

```
gcaacuuccuucguaagaaagguuacaggcggugauuguccaaacgcaauucuuguuuuauuguuauucgagaacuguguauggacaucagugcuugccggccgguuuc
......................................................................agaacCguguauggacaucagu.................  1     1  Fm3
......................................................................agaacuguguauggacaucaguA................  15    1  Fm3
......................................................................agaacuguguauggacaucaguU................  112   1  Fm3
......................................................................agaacuguguauggacaucagug................  1     0  Fm3
.......................................................................gaacuguguauggacauc....................  3     0  Fm3
.......................................................................gaacugugCauggacauc....................  1     1  Fm3
.......................................................................gaacuguguauggacauca...................  1     0  Fm3
.......................................................................gaacuguguaugUacaucag..................  1     1  Fm3
.......................................................................gaacuguguaAggacaucag..................  1     1  Fm3
.......................................................................gaacuguguauggacaucag..................  17    0  Fm3
.......................................................................gaacuguguauggacaucagu.................  83    0  Fm3
.......................................................................gaacuguguauggacGucagu.................  1     1  Fm3
.........................................................................acuguguauggacaucagug................  6     0  Fm3
..........................................................................cuguguauggacaucagA.................  1     1  Fm3
..........................................................................cuguguauggacaucagu.................  16    0  Fm3

...............................Cugauuguccaaacgcaauucu........................................................  1     1  Mm3
................................ugauuguccaaacgcaau...........................................................  1     0  Mm3
................................ugauuguccaaacgcaauuc.........................................................  24    0  Mm3
................................ugauuguccaaacgcaauucu........................................................  57    0  Mm3
................................ugauuguccaaacUcaauucu........................................................  1     1  Mm3
................................ugauuguccaaacgcaauucuu.......................................................  2     0  Mm3
................................ugauuguccaaacgcaauucuug......................................................  19    0  Mm3
..............................................................guuauucgagaacuguguauggacaucagu.................  1     0  Mm3
...............................................................uuauucgagaacuguguauggacauc....................  1     0  Mm3
.................................................................auucgagaacuguguauggacaucag..................  2     0  Mm3
.................................................................auucgagaacuguguauggacaucagu.................  1     0  Mm3
.....................................................................gagaacuguguauggacaucag..................  1     0  Mm3
.....................................................................Cagaacuguguauggacaucagu.................  5     1  Mm3
......................................................................agaacuguguauggacau.....................  1     0  Mm3
......................................................................agaacuguguauggacGuc....................  1     1  Mm3
......................................................................agaacuguguauggacauU....................  8     1  Mm3
......................................................................agaacuguguauggacauc....................  95    0  Mm3
......................................................................agaacuguguauggacauA....................  2     1  Mm3
......................................................................agaacuguguauggacaucU...................  7     1  Mm3
......................................................................agaacuguguauggacauca...................  195   0  Mm3
......................................................................agaacuguguauggacaucG...................  1     1  Mm3
......................................................................agaacuguguauggacCuca...................  1     1  Mm3
......................................................................agaacuguguauggacaAca...................  1     1  Mm3
......................................................................agaUcuguguauggacauca...................  1     1  Mm3
......................................................................agaacuguguauggacaGca...................  1     1  Mm3
......................................................................agaacuguguauggacaucaU..................  10    1  Mm3
......................................................................agaacuguguauggacCucag..................  2     1  Mm3
......................................................................agaacuguguCuggacaucag..................  1     1  Mm3
......................................................................agaacuguguauggacaCcag..................  9     1  Mm3
......................................................................Ugaacuguguauggacaucag..................  2     1  Mm3
......................................................................agaacCguguauggacaucag..................  1     1  Mm3
......................................................................agaacuguguauggacaucaC..................  1     1  Mm3
......................................................................agaacuguguauggacaucUg..................  1     1  Mm3
......................................................................agaacuguguauggacaucag..................  1085  0  Mm3
......................................................................agaacuguguauggacauUag..................  2     1  Mm3
......................................................................agaUcuguguauggacaucag..................  1     1  Mm3
......................................................................agaacuAuguauggacaucag..................  1     1  Mm3
......................................................................agaacuguguauggacaAcag..................  4     1  Mm3
......................................................................agaacuguguauggacaucaA..................  1     1  Mm3
......................................................................agaacuguguUuggacaucag..................  1     1  Mm3
......................................................................agaacuguguauggacGucag..................  2     1  Mm3
......................................................................agaacuguguauCgacaucag..................  1     1  Mm3
......................................................................agaacugAguauggacaucag..................  1     1  Mm3
......................................................................agaacuguguauggacaucGg..................  2     1  Mm3
......................................................................agaacuguguauggacUucag..................  5     1  Mm3
......................................................................agaacuguguauggacaGcag..................  4     1  Mm3
......................................................................agaacNguguauggacaucag..................  1     1  Mm3
......................................................................Ugaacuguguauggacaucagu.................  6     1  Mm3
......................................................................agaacuguguauggacaucagG.................  9     1  Mm3
......................................................................agaacugGguauggacaucagu.................  1     1  Mm3
......................................................................agaacuguguauggacaucaUu.................  5     1  Mm3
......................................................................agaacuguguGuggacaucagu.................  9     1  Mm3
......................................................................agaaGuguguauggacaucagu.................  2     1  Mm3
......................................................................agaacuguguauggacaucagC.................  6     1  Mm3
```

Star **Mature**

gcaacuuccuucguaagaaagguuacaggcggugauuguccaaacgcaauucuuguuuuauuguuauucgagaacuguguauggacaucagugcuugccggccgguuuc			
....agaacuguguauggGcaucagu.................	2	1	Mm3
....agaacuAuguauggacaucagu.................	1	1	Mm3
....agaaUuguguauggacaucagu.................	1	1	Mm3
....aCaacuguguauggacaucagu.................	1	1	Mm3
....agaacuCuguauggacaucagu.................	2	1	Mm3
....agaacuguguauggacCucagu.................	7	1	Mm3
....Ggaacuguguauggacaucagu.................	2	1	Mm3
....agaacugugCauggacaucagu.................	2	1	Mm3
....agaacuguguauggacaGcagu.................	20	1	Mm3
....agaacugugAauggacaucagu.................	1	1	Mm3
....agaacuguguauggacaucagu.................	5823	0	Mm3
....agaacuguguauggacGucagu.................	5	1	Mm3
....agaacuguguUuggacaucagu.................	7	1	Mm3
....agaacuguguauggacaCcagu.................	30	1	Mm3
....agaacuguguCuggacaucagu.................	5	1	Mm3
....aAaacuguguauggacaucagu.................	1	1	Mm3
....agaacuUuguauggacaucagu.................	1	1	Mm3
....agUacuguguauggacaucagu.................	1	1	Mm3
....agaacCguguauggacaucagu.................	1	1	Mm3
....agGacuguguauggacaucagu.................	5	1	Mm3
....agaacuguguauggacUucagu.................	6	1	Mm3
....agaacuNuguauggacaucagu.................	1	1	Mm3
....aUaacuguguauggacaucagu.................	1	1	Mm3
....agaacuguguauggacauUagu.................	5	1	Mm3
....agaUcuguguauggacaucagu.................	1	1	Mm3
....agCacuguguauggacaucagu.................	2	1	Mm3
....Ngaacuguguauggacaucagu.................	4	1	Mm3
....agNacuguguauggacaucagu.................	1	1	Mm3
....agaacugCguauggacaucagu.................	1	1	Mm3
....agaacAguguauggacaucagu.................	1	1	Mm3
....agaacuguguaugAacaucagu.................	1	1	Mm3
....agaacuguguauggaUaucagu.................	2	1	Mm3
....agaacuguguaAggacaucagu.................	1	1	Mm3
....agaacuguguauggacaAcagu.................	27	1	Mm3
....agaacuguguauggacauAagu.................	1	1	Mm3
....agaacuguguauggacaucagA.................	12	1	Mm3
....agaacuguUuauggacaucagu.................	3	1	Mm3
....agaacuguguauggacaucaguA................	6	1	Mm3
....agaacuguguauggacaucaguU................	24	1	Mm3
....agaacuguguauggacaucagug................	1	0	Mm3
....agaacuguguauggacaucagugU...............	2	1	Mm3
....gaacuguguauggacaucag..................	1	0	Mm3
....gaacuguguauggacaucagu.................	22	0	Mm3
....gaacuguguauggacaucaUu.................	1	1	Mm3
....gaacuguguauggacauAagu.................	1	1	Mm3
....aacuguguauggacaucagu.................	1	0	Mm3
....cuguguauggacaucagu.................	2	0	Mm3
